# Intelectual demand and formal education as cognitive protection factors in Alzheimer’s disease

**DOI:** 10.1590/S1980-57642010DN40400011

**Published:** 2010

**Authors:** José Roberto Wajman, Paulo Henrique Ferreira F. Bertolucci

**Affiliations:** 1 Neuropsychologist, Behavioral Neurology Section. Behavioral Neurology Section, Department of Neurology asnd Neurosurgery, Escola Paulista de Medicina, UNIFESP; 2 Neurologist, Behavioral Neurology Section, Department of Neurology and Neurosurgery, Escola Paulista de Medicina, UNIFESP.

**Keywords:** educational level, neuropsychological assessment, Alzheimer’s disease

## Abstract

To investigate the possible association between educational level and previous professional occupation, and objective cognitive and functional evaluation in a sample of elderly patients with Alzheimer’s disease.

**Methods:**

Through retrospective analysis of medical files, 174 patients with probable Alzheimer disease were randomly selected, classified and submitted to analysis according to previous professional occupation and years of formal education.

**Results:**

Subjects with lower education and less intellectually-demanding occupations performed worse than higher educated subjects in all cognitive subtests and on the functional scale.

**Conclusions:**

Results indicate that not only the total years of education, but also professional occupation has an impact on cognition and functioning in accordance with the hypothesis of cognitive reserve. Our findings confirmed this hypothesis, where subjects with higher education/ higher intellectual demand manifested first symptoms later than low education/ low intellectual demand subjects, with the latter group also exhibiting faster disease progression.

## Abstract (pt)

Analisar as possíveis correlações entre nível educacional e atividade profissional prévia, com dados objetivos de avaliação cognitiva e funcional em uma amostra de idosos com doença de Alzheimer.

**Métodos:**

A partir de análise retrospectiva de prontuário médico foram selecionados aleatoriamente 174 pacientes com provável doença de Alzheimer que foram subdivididos e submetidos a análise conforme sua atividade profissional prévia e nível de escolaridade alcançado formalmente, calculado em anos.

**Resultados:**

Em praticamente todos os subtestes cognitivos, além de escala funcional, os participantes do grupo menos escolarizado e profissionais de áreas consideradas menos complexas em relação a demanda intelectual, saíram-se pior quando comparados com grupo mais escolarizados.

**Conclusões:**

Não apenas o total acumulado de anos formais concluídos, mas também a atividade profissional previamente exercida parece ter resultado sobre a cognição e funcionalidade à medida que evolui a doença crônico degenerativa, favorecendo assim o conceito de reserva intelectual. Por esta hipótese espera-se retardo no início dos sintomas da doença em pessoas com alta escolaridade/ alta demanda intelectual e, por outro lado, a baixa escolaridade/ baixa demanda intelectual profissional podem estar associadas a início precoce e progressão mais rápida, tanto do ponto de vista cognitivo como funcional.

The aging of the human population is a worldwide phenomenon occurring at an ever faster pace. In 1950 there were 204 million elderly people. Five decades later, in 1998, this number had increased to 579 million, with a yearly increase of 8 million elderly. Projections estimate that by 2050 there will be 1900 million elderly, equivalent to the current world population of 14-year-olds. One explanation for this pattern is the worldwide increase since 1950 of 19 years in life expectancy at birth.^1^ According to forecasts, by 2050 the population group aged 100 years or older will increase 15-fold, from 145,000 to 2.2 million. In Brazil centenarians numbered 13,865 in 1991, and had increased by 77% to 24,576 by 2000. São Paulo state has the highest number of centenarians (4,457), followed by Bahia (2,808), Minas Gerais (2,765) and Rio de Janeiro (2,029) States. According to a national dwellings sample census (2008) there are 21 million elderly in Brazil. Average formal education for this group is 4.1 years, distributed as follows: no education or less than one year (32.2%); one to three years (19.5%); 4 to 8 years (31.3%); 9 years or above (17.1%).

While several investigations consider literacy (basic ability to read and write) as synonymous to education, the correlation is weak, and often there is discordance between the definition and its meanings.^2,3^ As an example, in an investigation involving an elderly population, mean formal education was 12 years, but literacy level was similar to that of 5^th^ grade students.^4^

“Literacy” and its antonymous “illiteracy” were coined in the thirties in United States and used in Second World War by the American Army to indicate ability to understand written instructions necessary to execute military tasks. These expressions have subsequently been used to indicate the pragmatic use of reading and writing in home and work daily settings, often set against academic education. In some cases, the expression “functional illiteracy” has been used to indicate a status between absolute illiteracy and partial grasping of rudimentary abilities necessary for “survival in industrial societies”.^5^

The ample dissemination of the expression “functional illiteracy” was due basically to a Unesco action that adopted it in the definition of literacy in 1978 to set a standard for education statistics and education strategies among member countries. Unesco’s definition of literacy proposed in 1958 referred to the ability to understand written texts or to write a short and simple paragraph on a daily life subject.

Twelve years after Unesco indicated another definition, defining literacy as “functional’ when sufficient for adequate integration of a subject into his/her environment, integration which included doing activities for which reading, writing or calculation are necessary.

In parallel with these new definitions there was an increase in life expectancy and an increase in the group of elderly people with neurological degenerative disorders with functional and cognitive decline.

The main objective of this investigation was to analyze the possible correlation between educational level and professional activity, and cognitive performance of a sample of Alzheimer disease subjects followed at a dementia outpatient clinic in the city of São Paulo.

## Methods

### Subjects

A systematic analysis of demographics, clinical data and neuropsychological evaluation of the medical files of 174 elderly people with a diagnosis of probable Alzheimer disease was conducted in accordance with the Research Ethics Committee guidelines. Diagnosis of probable Alzheimer disease was reached according to DSM-IV criteria.^6^ All participants were on medication (class of anticholinesterasic), without other neurological conditions, psychiatric or cerebrovascular disease history, and were followed at the Behavioral Neurology Section of the Universidade Federal de São Paulo in 2010.

### Study design

Subjects included in this study were classified and divided into groups according to educational level in formal years and previous professional activity from which they retired and in which they worked longest. Stratification with regard to previous occupation was performed such as into farm work, for any rural activity (cattle raising or vegetable cultivation). Similarly technical jobs at the same level were included in the same group.

For statistical analysis, two groups were compared: Group I – subjects classified as commerce workers, with middle or higher (University) education; Group II – rural and construction workers, and household domestics. After recording gender, age, formal education level and estimated time of disease duration, subjects were submitted to the Clinical Dementia Rating (CDR),^7^ MMSE^8^ and specific sub items of the neuropsychological battery^9^ (direct and inverse digit span, total score for immediate recall for three trials of a 10-word list, delayed recall of the same list, naming of 15 drawings from the Boston naming test and verbal fluency for animals).

### Statistical methods

Descriptive analysis of all variables was carried out. For quantitative variables this was done using lowest and highest scores, mean and standard deviation. For qualitative variables, absolute and relative frequencies were calculated. On comparison between groups, normality of data distribution was rejected and the Mann-Whitney non-parametric test^10^ was therefore used. To test homogeneity between proportions, the Chi-square test^10^ or Fisher exact test^10^ (when expected frequencies were lower than 5) was used. Significance level was set at 5%.

## Results

**Figure 1:**
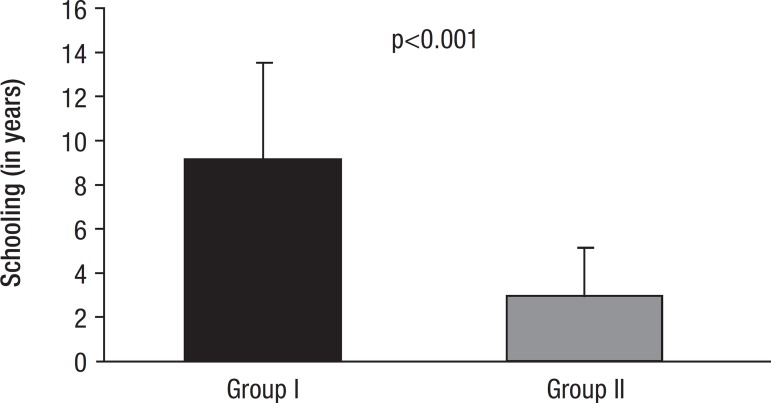
Educational level.

No difference in age (Group I – 74.35 years; Group II – 74.46 years) was found between the two groups. Differences in education between Groups I and II are shown in Figure 1 and reached significance for Group I (9.10±4.38) vs Group II (2.99±2.13). Comparing this variable with others revealed a correlation with all tests and with disease duration (Group I – 3.28±1.71; Group II – 2.52±1.35).

Table 1 shows a difference for gender in Group II, which contained more females, while no difference was seen in Group II.

**Table 1:** Distribution according to gender, CDR and previous occupation.

	Class	Groups				
		I			II	
		N	%		N	%
Gender	F M	25 35	41.7 58.3		85 29	74.6 25.4
CDR	1 2 3	37 20 3	61.7 33.3 5.0		40 56 18	35.1 49.1 15.8
Profession	Farming Building Home made House wife Graduation Technical Trading	0 0 0 0 13 15 32	0.0 0.0 0.0 0.0 21.7 25.0 53.3		14 21 22 57 0 0 0	12.3 18.4 19.3 50.0 0.0 0.0 0.0

**Figure 2:**
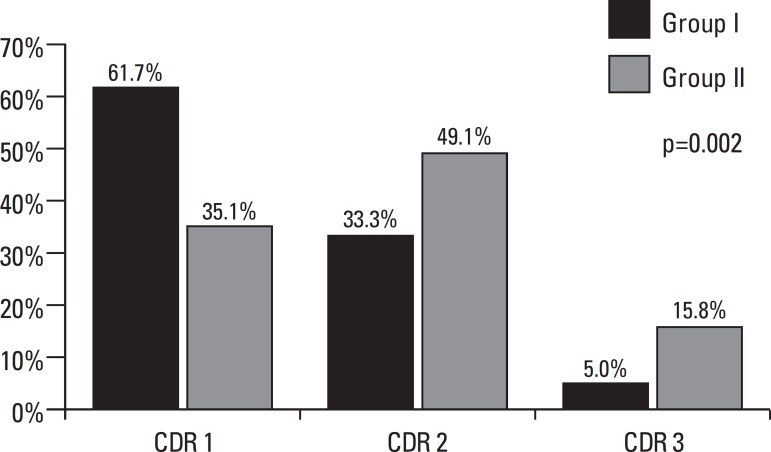
Disease stage (CDR) for Groups I and II .

In terms of demographic and clinical data there was a higher incidence of moderate to moderately-severe disease in Group II (Figure 2). For Group I, most subjects were at an early stage of disease, while for Group II most subjects were at a moderate stage AD.

Raw scores presented at Table 2 show that except for immediate recall there was a significant difference for all tests, from screening (MMSE) to attention, working memory, and language. Comparisons Group I subgroups (commerce workers, technical jobs and University graduated people), using Kruskal-Wallis test, showed no difference for neuropsychological performance, CDR and disease duration (Table 3).

**Table 2:** Age, education, disease duration and neuropsychological performance.

	Group	N	Average	sd	Median	Minimo	Maximum
Age	III	60114	74.3574.46	7.646.78	75.0074.50	5760	9694
Schooling	III	60114	9.102.99	4.382.13	8.004.00	30	1610
Disease duration (in years)	III	60114	3.282.52	1.711.35	3.002.00	11	108
MMSE	III	60114	20.3515.96	5.055.10	21.0015.50	62	2927
Span F	III	60114	5.474.67	1.281.23	6.005.00	30	89
Span B	III	60114	3.001.94	1.151.12	3.002.00	00	64
Total list	III	60114	10.238.00	3.584.10	10.008.00	20	2017
Evocation	III	60114	1.050.89	1.441.34	0.000.00	00	65
SVF	III	60114	8.637.35	3.633.07	8.007.00	01	1815
Boston 15 items	III	60114	11.358.89	3.073.29	12.009.00	20	1515

**Table 3:** Group I subgroups comparison.

	Profession	N	Average	sd	Median	Minimo	Maximum
Disease duration (in year)	TradingGraduationTechnical	321315	3.003.543.67	1.681.272.06	333	122	8610
CDR	TradingGraduationTechnical	321315	1.411.621.33	0.560.770.49	111	111	332
MMSE	TradingGraduationTechnical	321315	20.8419.6219.93	5.394.375.04	212121	6149	292528
Span B	TradingGraduationTechnical	321315	3.032.693.20	1.231.180.94	333	002	645
Total list	TradingGraduationTechnical	321315	10.759.469.80	3.902.733.53	10108	247	191420
SVF	TradingGraduationTechnical	321315	8.667.699.40	3.244.423.74	878	304	151318

CDR: Clinical Dementia Rating; MMSE: Mini-Mental State exam; B: Digit Span Backward Total List: Total of words recalled from CERAD word list (0-30); SVF: Semantic Fluency Verbal for animals.

## Discussion

The relationship between dementia and more specifically, Alzheimer’s disease, and education has been investigated since last decade, with largely concordant results. In line with previous studies,^11-14^ the findings of this investigation showed that low education and less cognitively-demanding activities are a significant factor for high incidence and earlier manifestation of this disease. By contrast, the combined accumulation of educational experience and use of cognitively more complex abilities might strengthen the cognitive reserve and preserve both cognition and functionality.

There are suggestions that early intellectual demand, necessary for formal education and later for professional occupation, may be crucial in the formation of cognitive resources over the long term and these resources may persist even following the onset of dementia.^15^ Evidence favoring this hypothesis is the objective demonstration, using neuropsychological tests, that those with lower formal education have more rapid decline following the first symptoms of dementia.^16^ This study revealed that, according to educational level, individuals with more years of formal studies had a delayed onset of symptoms and that ,from diagnosis, cognitive decline was less marked in this group.

Despite increasing evidence favoring this hypothesis (for a review see Paradise et al.^17^), not all less educated individuals or those working in less demanding activities will have an earlier death, thus indicating that survival might be related to other factors other than cognitive reserve.

Our investigation identified three classes of patients with three different levels of education and activity, that had equivalent education and activity, with regard to first symptoms of dementia and neuropsychological performance. However, the authors note that more data are needed to establish which factors are actually protective against early appearance and manifestation of neurodegenerative disease, along with studies including specific statistical regression analysis of clinical and sociodemographic variables.
